# Reprogramming—Evolving Path to Functional Surrogate β-Cells

**DOI:** 10.3390/cells11182813

**Published:** 2022-09-08

**Authors:** Eric Kalo, Scott Read, Golo Ahlenstiel

**Affiliations:** 1 Blacktown Clinical School and Research Centre, School of Medicine, Western Sydney University, Blacktown, NSW 2148, Australia; 2 Blacktown Hospital, Blacktown, NSW 2148, Australia; 3 Storr Liver Centre, The Westmead Institute for Medical Research, University of Sydney, Westmead, NSW 2145, Australia

**Keywords:** β-cell, diabetes mellitus, cell sources, reprogramming, insulin

## Abstract

Numerous cell sources are being explored to replenish functional β-cell mass since the proof-of -concept for cell therapy of diabetes was laid down by transplantation of islets. Many of these cell sources have been shown to possess a degree of plasticity permitting differentiation along new lineages into insulin-secreting β-cells. In this review, we explore emerging reprograming pathways that aim to generate bone fide insulin producing cells. We focus on small molecules and key transcriptional regulators that orchestrate phenotypic conversion and maintenance of engineered cells.

## 1. Introduction

Over the past years, the global incidence of diabetes mellitus has dramatically increased alongside the obesity epidemic. With more than 537 million people affected by the disease globally, it is projected to increase to more than half a billion by 2030 [1]. It is becoming an ever-growing burden threatening to overwhelm many health care systems and economies throughout the world [2].

Diabetes is described as a group of heterogeneous metabolic diseases characterized by common elements of hyperglycemia and glucose intolerance due to defects in insulin section, impaired effectiveness of insulin or both. In the progression of both type 1 diabetes mellitus (T1DM) and type 2 DM (T2DM), there comes a point where a threshold percentage of β-cells become dysfunctional, leading to a reliance on exogenous insulin administration for the treatment of patients with DM.

Although the isolation of insulin in 1921 marked a panacea that has drastically transformed diabetes from a terminal to a treatable illness, the precise temporal glucose control supplied by endogenous insulin-producing β-cells is not matched by the current methods of insulin delivery. Exogenous insulin therapy, as a result, can pose a risk of hypoglycemia that can lead to life threatening coma and premature death [3]. Consequently, intensive insulin therapy targeting near-euglycemia can prevent the risk of long-term microvascular and macrovascular complications. Data from large population studies have consistently supported the notion that tight glycemic control can effectively reduce the development of diabetes complication and conferred mortality and morbidity benefits [4,5,6].

Continuous glucose monitoring and exogenous insulin administration are considered the mainstay of treatment for diabetes. While it significantly leads to improvement of patient outcomes, it falls short in achieving optimal long-standing blood glycaemia control, especially for T1DM patients [7,8,9]. In a recent randomized, multicenter trial, results showed that the percentage of time that blood glucose remained within the target glycemic range (notwithstanding automated insulin delivery systems) was persistently suboptimal in patients with T1DM [10]. Consequently, therapies that result in perpetual reconstitution of a physiological blood glucose setpoint are an extremely sought approach in the long term.

Rather than modestly alleviating both type 1 and type 2 diabetes, β-cell replacement has the potential to reverse these conditions. A successful pancreas transplant can provide a closed-loop system that restores euglycemia without the risk of severe hypoglycemia, thus halting the development or progression of complications. Nonetheless, major surgery associated risks, chronic shortages of donor pancreata, graft rejection and the burden of life-long immunosuppression regimens set the utility of such an approach exclusively to a narrow group of uremic patients with brittle diabetes.

To overcome the need for major surgery associated with pancreas transplantation, research has focused on the development of protocols to isolate pancreatic islets. Despite the outstanding success of islet of Langerhans transplantation in lowering insulin dependence for years in patients with T1DM, the widespread application of this approach has been hampered by the scarcity of donors, as well as immunological challenges that are further aggravated by toxic effects of long-term immunosuppressive drugs [11]. Notwithstanding the pitfalls, the success of this approach has fueled efforts for a generation of bone fide functional β-cell mass that maintains the physiological oscillation of insulin secretion and responsiveness to dysregulated glycemia. Moreover, recent studies using autologous cell therapies may halt the requirement of lifelong immunosuppressive therapy.

Here, we review cell sources, reprogramming tools and the emerging paths envisioned in the generation of functional β-cell mass for diabetes mellitus cell therapy.

## 2. Reprogramming Cells to Make Insulin

For the last 20 years, significant efforts have focused on the generation of human embryonic stem cells, hESC-derived β-cells, that exhibit sustainable insulin secretion features, which mainly mirror the dynamicity of native human islets. By replicating the signaling events and physiologically relevant cues that dictate fate transition through definitive endoderm and pancreatic endoderm to hormone-expressing cells during human pancreas development, β-like cells can be produced [12,13,14]. The particular interest in hESCs is supported by the fact that these cells have the capacity of extensive self-renewal and virtually can be differentiated into derivatives of all three germ layers.

Numerous studies have outlined differentiation protocols of insulin-producing cell types from hESCs [15,16,17,18,19,20,21]. However, reconstructing functional equivalent, mono-hormonal cells producing insulin under cell culture conditions has been intangible. This is likely because the in vitro systems utilized lack critical signals present in vivo, including the close interaction between exocrine, ductal and endocrine cells [15,20,22,23]. Among the critical concerns with hESC-derived therapeutic products is the occurrence of unwanted cell populations during in vitro differentiation that might interfere with the activity of the desired cell populations. Another concern is the risk of tumorigenicity. Moreover, the wider use of ESCs for research reasons is still largely hampered by numerous governmental bans concerning the use of human embryos in many countries, as well as by ethical and religious sensitivities.

**Figure 1:**
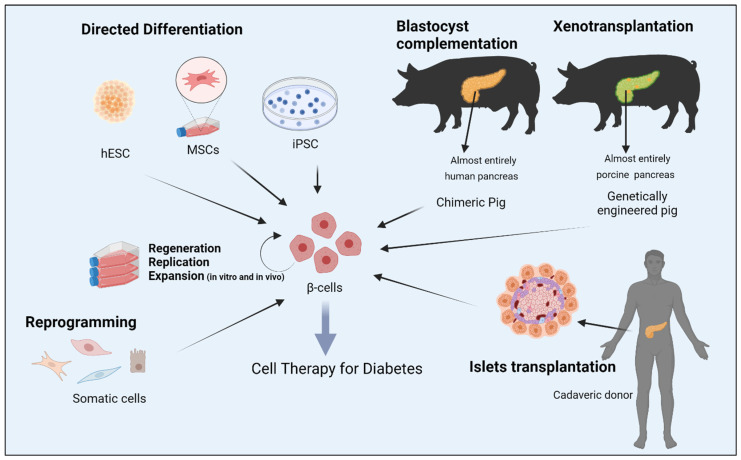
Strategies aimed at generation of bone fide β-cell for replacement therapy in diabetes. Abbreviations: human embryonic stem cells (hESC); mesenchymal stem cells (MSCs); induced pluripotent stem cells (iPSC). Figure was created with BioRender http://www.biorender.com.

Compared to ESC and induced pluripotent stem cells (iPSCs), adult stem cell-like, mesenchymal stem cells (MSC) are considered ideal candidates to generate functional beta cells for personalized medicine owing to their superior anti-inflammatory, immunomodulatory and angiogenic properties. Numerous studies have shown that MSCs isolated from ample tissues and organs, such as bone marrow, adipose tissue, Wharton’s jelly, umbilical cords matrix blood, placenta and dental pulps, possess a developmental plasticity to differentiate into functional insulin producing cells with similar cytoarchitecture and functionality to β-cells. Consequently, utilizing MSC transplantation for the treatment of diabetes has been the focus of randomized controlled trails (RCTs) for the last few years. Although several clinical trials have shown that MSCs can reduce hyperglycemia by increasing insulin secretion in humans, the lack of control arms in some small sample sizes, inconsistent methods of isolation and delivery of MSCs, adverse effects and the failure to sustain therapeutic effect longitudinally from MSC therapy were common limitations in almost all RCTs. Finally, genetically modified animals designed for xenotransplantation or interspecies chimera-derived human organs, by using the blastocyst complementation method, could potentially offer unlimited sources of β-cells. Figure 1 summarizes several strategies aimed at generation of bone fide β-cell for replacement in diabetes.

## 3. Alpha to β-cell Reprogramming

A logical place to begin with for generating β-cells is to utilise the plasticity of closely related endoderm derived cell types like pancreatic non-β-cells and coaxing them to adopt a β-cell phenotype. Given the close ontogenetic relationship, functional similarity and dependency among these cells, the potential for interconversion is unequivocal [24]. Phenotypic plasticity between pancreatic α-cells and β-cells is notably pronounced. A prominent study several years ago demonstrated that inter-endocrine plasticity and proliferation of β-cells can be elicited upon increased metabolic demand or after substantial diphtherial toxin induced β-cell loss [25]. While this study was performed in mice, there is no evidence to suggest that a similar intrinsic cell type interconversion event can occur in other settings of β-cell damage in humans. Of note, a similar phenomenon of β-cell loss may naturally occur in mice transitioning into adulthood from puberty.

Forced overexpression of key transcriptional regulators, the paired/homeodomain transcription factor (PAX4) [26], Insulin promoter factor 1 (PDX1) [27], or PDX1 and MAF BZIP Transcription Factor A (MAFA) [28], inhibition of DNA (cytosine-5)-methyltransferase 1 (DNMT1) and Aristaless related homeobox (ARX) [29], have also been shown to successfully drive α- to β-cell conversion. Yang et al. illustrated that PDX1 alone is not sufficient to allow the full reprogramming of glucagon-expressing cells [27]. This points to a chronotypic effect, where a subset of endocrine progenitors is primed for fate switch at the peri/postnatal stage, highlighting the role of the epigenome in reprogramming events. Importantly, the applicability of these findings to human cells needs to be determined, given that most of these studies were conducted in rodents. A subsequent study sought to better study human islet cell plasticity by lineage tracing and reprogramming α-cells with Mafa and Pdx1. Remarkably, insulin-producing α-cells retained α-cell markers, as evidenced by transcriptomic and proteomic characterization, while retaining insulin production and reversing diabetes for 6 months [30]. Several studies highlighted the type of pancreatic gene delivery systems for efficient intrinsic alpha cell transduction. Using adeno-associated virus (AAV) rather than adenoviral or lentiviral vectors has been shown to exhibit long-term gene expression induced reprogramming of pancreatic α-cells into functional β-cells [31].

The development of mature pancreatic endocrine cell subtypes represents the culmination of complex fate determining transcriptional programs that orchestrate the transition from one progenitor state to another. As transcription factors are sufficient to induce α- to β-cell reprogramming, it will be imperative to identify molecules that govern such a conversion as they would permit better control of the process [32]. For example, α-cells treated with glucagon-like peptide receptor agonists, exendin-4, can enhance pancreatic α-cells proliferation and their trans-differentiation into β-cells [33]. Similarly, sustained γ-Aminobutyric acid (GABA) exposure can induce concomitant α-cell to β-like cell neogenesis in vivo from human islet α-cells transplanted in mice by mobilization of duct-lining precursor cells that adopt an α cell identity [34]. GABA is a critical paracrine signal released by β-cells that inhibits glucagon-release from α-cells [35]. Artemisinin, a class of antimalarial agents, has been shown to impair α-cell identity in immortalized rodent cell lines by enhancing GABA receptor signaling, and by functionally inhibiting the ARX transcription factor [36]. In contrast, other studies have found that inhibition of ARX after long-term treatment by artemether, a derivative of artemisinin, did not furnish any α- to β-cell trans-differentiation in primary mouse islets, suggesting that these regulators may not represent a viable route to a novel diabetes therapy [37,38]. Nonetheless, in support of glucagon inhibition as a mechanism of α- to β- cell transition, the monoclonal antibody antagonist of the glucagon receptor (Ab-4) has been shown to enhance the formation of functional β-cell mass and their conversion from α-cell precursors in a rodent model of T1DM [39].

## 4. Pancreatic Non-Endocrine Cells

### 4.1. Acinar Cell Reprogramming

Acinar cells are the most abundant exocrine cell type in the pancreas. In contrast to ductal cells, acinar cells and endocrine islet cells share a common progenitor occurring after separation from the duct cell lineage. Like α-cells, pancreatic acinar cells retain a degree of plasticity that enables a change in their differentiation commitment. These cells can undergo acino-ductal trans-differentiation, allowing them to be pushed back to a progenitor state [40]. Acinar cells lose key digestive characteristics and gain embryonic Notch signaling and ductal characteristics, including the expression of transcription factor Pdx1, neuroendocrine marker PGP9.5 and cytokeratins CK-19 and CK-20.

The rationale behind utilizing acinar cells for de novo β-cell generation stems from the observation of single β-cells becoming scattered throughout the exocrine parenchyma in T1DM patients after immunosuppressive therapy [41]. In addition, various studies have been able to differentiate acinar cells into a β-cell like phenotype in vitro [42]. Like α- to β-cell differentiation, numerous methods have been adapted to acinar cells. Adenovirus-mediated delivery of key β-cells genes PDX1, MAFA and Neurogenin-3 (NGN3) [PMN cocktail], for example, can generate β-cell like cells in mice [43]. Moreover, encouraging results from experiments with human acinar cells have shown that the addition of Pax4 to the PMN-cocktail, in addition to ARX suppression, resulted in β-cells differentiation, albeit with a low conversion rate [44]. Improving methods of transcription factor delivery and reprogramming protocols will surely improve yields of glucose-responsive, insulin-secreting cells from acinar cells. Access to the target acinar cells via clinical tools such endoscopic retrograde cholangiopancreatography (ERCP) may improve the application of transcription factors or their vectors (e.g., adenovirus) into the pancreatic ducts, thus improving the rate of cell trans-differentiation [45]. Lastly, the addition of epidermal growth factor (EGF) and leukemia inhibitory factor (LIF) to β-cells differentiated from adult pancreatic exocrine cells has been shown to render them less susceptible to cytokine induced cell-death [46]. Such additional treatments have the potential to improve cell yields following ex vivo culture, whilst enabling cells to develop into islet-like structures [47]. 

### 4.2. Duct Epithelial Cells

The other endodermal cell population in the pancreas, namely, the ductular epithelium, is another potential candidate for reprogramming. The concept of reprogramming these cells has evolved from embryonic development, where endocrine cells are developed from budding ductal wall. In line with this, throughout the pancreas, islets adjacent to exocrine ducts ubiquitously exist. Moreover, during conditions of high secretory demands in rodents, such as following a pancreatectomy, increased numbers of ducto-insular complexes are typically observed [48,49].

Several groups have reported the presence of endocrine progenitor cells within the ductal epithelium, that serve as a pool for both islet and acinar tissues after birth and into adulthood [50,51]. Indeed, expanded human ductal cells transplanted into immunocompromised mice have the ability to generate insulin-positive cells [52,53]. High levels of PDX1 expression were reported in human pancreatic ducts, suggesting additional similarities between islets and ductal cells [54]. Consistent with these findings, grafts of purified human duct cells can harbor insulin positive cells expressing duct markers [55]. Numerous markers for these ductal progenitor populations have been identified, including CD133, CD49fhigh, DCLK1 and ALDH1+ [56,57,58,59,60]. 

Several strategies can facilitate the process of trans-differentiation from duct cells into β-cells. In line with other approaches of stem cell activation, β-cell progenitors within ductal lining can initiate differentiation in an Ngn3-dependent manner upon pancreatic injury in the adult rodent pancreas [61,62]. Because NGN3 is the earliest islet cell-specific transcription factor in embryonic development, various other methods to express a set of genes that are characteristic for endocrine cells of pancreatic islets can be exploited for the induction of cultured human ductal cells. Ngn3 overexpression, combined with modulation of the Delta-Notch signaling, the addition of pancreatic endocrine transcription factors (Myt1, Mafa and Pdx1) [63], or either treatment with Activin A and Exendin 4 with high glucose, may lead to activation of transcription factors necessary for inducing endocrine phenotype [64]. Transgenic hepatic overexpression of the cytokine TWEAK (TNF-like weak inducer of apoptosis, TNFSF12) also promotes transient expression of Ngn3, resulting in β-cells derived from pancreatic duct epithelium [65]. Lastly, critical inflammatory cytokines implicated in the pathogenesis of diabetes (TNF-α, IL-1β and IFN-γ) have been demonstrated to stimulate NGN3 activation in a STAT3-dependent manner the endocrine differentiation program in duct cells [66].

Other strategies that have demonstrated duct-to-β cell progression include pancreatic or ductal deletion of the Fbw7 component of the SCF-type ubiquitin ligase [67] and Pax4 ectopic expression in glucagon-positive cells [26]. Varying success with in vitro differentiation of human islet-depleted exocrine tissue (both duct and acinar cells) or duct cell lines with BMP7, gastrin and epithelial growth factor (EGF) [68], or preadipocyte factor 1 (Pref1), have also been reported [69,70,71].

Enhancing ductal trans-differentiation into insulin producing cells is hampered by the dangers of gene editing technologies. Infection of adult and human duct cells with adenoviral vectors may bypass these issues by expressing Pdx1, Ngn3, neurogenic differentiation 1 (NeuroD1) or Pax4, which have also been shown to induce insulin gene expression [72]. Moreover, the use of the protein transcription domain from NEUROD1 can permeate pancreatic islets, due to its arginine- and lysine-rich protein transduction domain sequence [73].

**Figure 2:**
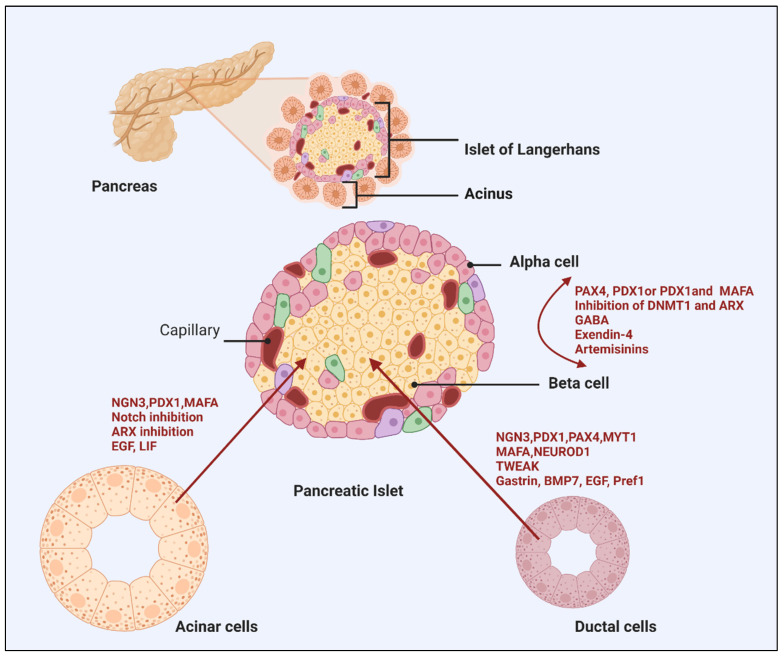
Trans-differentiation paths of differentiated pancreatic cells into functional β cells. Abbreviations: Aristaless related homeobox (ARX); DNA (cytosine-5)-methyltransferase 1 (DNMT1); Epidermal growth factor (EGF); Insulin promoter factor 1 (PDX1); Leukemia inhibitory factor (LIF); MAF BZIP Transcription Factor A (MAFA); Neurogenin-3 (NGN3); Neurogenic differentiation 1 (NEUROD1); the paired/homeodomain transcription factor (PAX4); Preadipocyte factor 1 (Pref1); TNF-like weak inducer of apoptosis, TNFSF12 (TWEAK); γ-Aminobutyric acid (GABA). Figure was created with BioRender http://www.biorender.com.

In conclusion, although data suggest that ductal cells could be used for trans-differentiation, not all studies support this notion. Additional evidence is required to demonstrate the robustness of this approach. Figure 2 summarises trans-differentiation paths of differentiated pancreatic cells into functional β cells.

## 5. Extra Pancreatic Cell Sources

**Figure 3:**
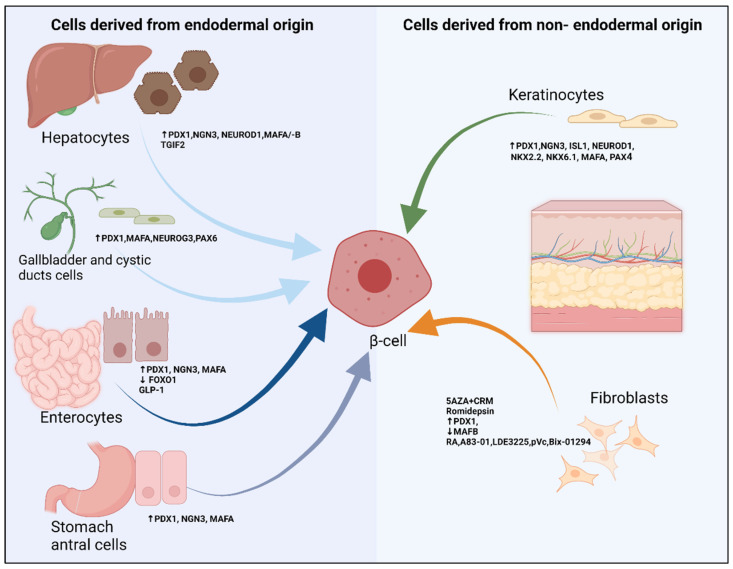
Extra pancreatic cell sources for generation of de novo β-cells. Abbreviations: Chromatin remodeling medium(CRM); Forkhead Box O1 (FOXO1); Glucagon-like peptide-1 (GLP1); Insulin Gene Enhancer Protein (ISL1); Insulin promoter factor 1 (PDX1); MAF BZIP Transcription Factor A (MAFA); MAF BZIP Transcription Factor B (MAFB); Neurogenin-3 (NGN3); Neurogenic differentiation 1 (NEUROD1); Homeobox protein Nkx2.2 (Nkx2.2); Homeobox protein Nkx6.1 (Nkx6.1); the paired/homeodomain transcription factor (PAX4); Paired box domain 6 (PAX6); TGFβ-induced factor homeobox 2 (TGIF2); 5-AZA 5-azacytidine. Combination of four small molecules (RA (Retinoic acid), A83-01 (a TGF-β receptor inhibitor), LDE225 (a hedgehog pathway inhibitor), and 2-phospho-L-ascorbic acid (pVc), and selective G9a and GLP inhibitor of histone methyltransferase (Bix-01294)) are small molecules that enhance generation of pancreatic progenitor-like cells. Figure was created with BioRender http://www.biorender.com.

Using extra-pancreatic cells as a source for insulin producing precursor cells has many advantages, including ease of autologous cell replacement, as well as the abundance of cell material, as compared to pancreatic tissue (Figure 3).

**(A)**  **Cells derived from endodermal origin**

### 5.1. Liver, Gallbladder and Cystic Derived Cells

Hepatic cells were among the first cell sources to be attempted for reprogramming. As pancreas and liver hepatocytes developmentally evolve from the same bi-potential precursors in the anterior endoderm in the embryonic foregut, it is natural to hypothesize that two closely related tissues may be inter-convertible. In addition, a subpopulation of oval cells with hepatocyte markers (a proposed hepatic progenitor population) has been found to reside within pancreatic tissue [74,75]. Purified cultures of these cells were reported to transdifferentiate into insulin producing cells in vitro, when cultured in the absence of glucocorticoids and upon long term exposure to high glucose concentration (similar to conditions permitting “pancreatic stem cells” to differentiate into insulin producing cells) [74].

Ectopic overexpression of PDX1 has been broadly investigated in mice and cultured hepatocytes as a tool for trans-differentiation of hepatocytes into β-cells. Ferber et al., as early 2000, reported in vivo reprogramming of mice liver cells into pancreatic β like cells, using recombinant-adenovirus-mediated gene transfer of PDX1 [76]. Likewise, immortalized human fetal liver progenitor cells have been successfully reprogrammed with lentiviral vectors harboring PDX1 into partial β-cell phenotype. An analogous approach using injections of helper-dependent adenoviral vectors demonstrated that expression of both betacellulin and NEUROD in hepatocytes can ameliorate hyperglycemia in a streptozotocin-induced diabetes mellitus murine model [77]. On the contrary, several studies suggested that ectopic PDX1 expression alone would not be sufficient to convert liver cells into pancreatic insulin-producing cells, and may in fact cause severe hepatitis in rodent liver and even liver dysmorphogenesis [78,79]. The contradictory results could be explained by the lack of the selective switch of PDX1-induced liver-to-pancreas. In line with the close lineage association between hepatocytes and pancreatic cells, liver-to-pancreas fate conversion can be elicited by induced expression of TGFβ-induced factor homeobox 2 (TGIF2), both ex vivo and in vivo. Moreover, it can serve as a liver fate decision versus developmental regulator of the pancreas [80].

Lastly, other tissues of the extrahepatic biliary tree, including the gallbladder and cystic duct, are susceptible to trans-differentiation into insulin producing phenotype by the overexpression of hallmark pancreatic endocrine transcription factors Pdx1, Mafa, Neurog3 and Pax6, and differentiation culture in vitro [81].

### 5.2. Intestinal and Antral Stomach Cells

Intestinal crypts and antral stomach niches are abundant in endocrine cells that retain a high degree of functional similarity to pancreatic β-cells [82,83]. Nearly a hundred years ago, soon after the discovery of secretin, the search for intestinal factors regulating the endocrine secretion of the pancreas began. The concept of entero-insular axis is entirely based on incretins [84,85,86]. The actions on β-cells of known incretins, including glucose-dependent insulinotropic polypeptide, also known gastric inhibitory peptide (GIP), and glucagon-like peptide-1 (GLP-1), largely intersect. Incretin actions include β-cell growth (anti-apoptotic action of incretins), modulation of glucose-stimulated insulin secretion, and enhanced insulin action [87,88,89]. Suzuki et al. demonstrated that in both neonatal and adult rats, intestinal epithelial cells when injected intraperitoneally with GLP-1, became glucose-responsive, insulin secreting cells capable of reversing diabetes [90]. The transcriptional activator PDX1 is known to stimulate insulin secretion in both β-cells and intestinal epithelia. Koizumi et al. showed that when pancreatic epithelia concurrently stimulated with exogenous GLP-1 and transduced with PDX1, they convert into bioactive insulin-secreting cells. The same group has shown that intestinal epithelia obtained from murine ileal loops, when transfected with PDX1, can express insulin, though they presented no data about the addition of GLP-1 to same cells [91].

More recently, a report by Chen et al. demonstrated that rapid conversion of intestinal crypt cells into endocrine cells can be achieved by transient intestinal expression of PDX1, MAFA and NGN3 (PMN cocktail), which coalesce into “neo-islets” below the crypt base. Neo-islet cells exhibited ultrastructural features of β-cells and were capable of insulin secretion. Importantly, intestinal neo-islets were shown to be glucose-responsive and able to ameliorate hyperglycemia in a diabetic murine model. These data are supported by human intestinal organoid studies, where PNM expression led to the conversion of intestinal epithelial cells into β-like cells [92]. Another report emphasises that the ablation of Foxo1 in NGN3 enteroendocrine progenitor cells give rise to gut insulin positive cells that convey mature β-cell characteristics [93].

Another tissue source highly amenable to reprogramming is stomach neuroendocrine cells. In streptozotocin-induced diabetic mice, it is either in vitro bioengineering of antral gastric cells, or in vivo reprogramming of antral gastric cells with the PMN cocktail into insulin secreting organoids can reverse hyperglycemia [94].

**(B)**  **Cells derived from non-endodermal origin**

### 5.3. Fibroblasts

Fibroblasts can undergo reprogramming into different cell types through various forms of genetic manipulation, but creating functional cells that respond to physiological stimuli has always been challenging. An important aspect dictating the utility of these cells is whether the age, or whether developmental timing can be rest by direct conversion, heterogeneity, or proliferative capacity of the starting population, will have an impact on the generation of β-cells.

A body of evidence suggests that dermal fibroblasts are a heterogeneous population and are distinct cell types that contain cells with variable differentiation potential [95,96]. Manipulating the ground state of the starting population may help overcome a major and thus far unsolved obstacle in trans-differentiation; in particular, why young donors can be efficiently converted than those from adult cells and may allocate the hierarchic model of inter-germ layer barriers [97].

Pennarossa et al. described a chemical method for reprogramming pig and human dermal fibroblasts [98,99]. A brief exposure of adult primary cells to 5 azacytidine (5-AZA) for 18 h to inhibit DNA methylation, immediately followed by a specific induction protocol, lead the conversion of 35% of fibroblasts into insulin producing cells. It is believed that treatment with 5-AZA in “chromatin remodeling medium” (CRM) can facilitate the transition of cells into a higher plastic state, but not enough to achieve stable pluripotency. The authors suggest that this renders inter-lineage conversion much closer to the physiological induction process that occurs during embryonic development.

Altering the epigenetic signature of cells using two compounds such as romidepsin, a histone deacetylase inhibitor, and 5-AZA, Katz LS et al. showed that human dermal fibroblasts display a distinctive regulation of expression of transcription factors involved in induction of glucagon and insulin as well as islet growth [100]. Overexpression of PDX1 and silencing of MAF BZIP Transcription Factor B (MAFB) resulted in a stronger induction of insulin as compared to romidepsin and 5-AZA alone. The cells derived from such a protocol are responsive to glucose-stimulated insulin secretion (GSIS) and can reduce blood glucose levels in diabetic mice.

Another paradigm of the trans-differentiation of fibroblasts was devised in which transient expression of pluripotency reprogramming factors in mouse embryonic fibroblasts, in conjunction with a unique combination of soluble molecules to produce functional endoderm-like progenitor cells, without passing first through pluripotency state. These definite endoderm-like cells can then be directed towards hepatic and pancreatic lineages. Small molecules combination (RA (Retinoic acid), A83-01 (a TGF-β receptor inhibitor), LDE225 (a hedgehog pathway inhibitor), and 2-phospho-L-ascorbic acid (pVc), and selective G9a and GLP inhibitor of histone methyltransferase (Bix-01294)) can further facilitate reprogramming of these cells. The resulting pancreatic progenitor-like cells could in vivo mature into functional, insulin-secreting β-like cells. It is unclear whether these findings from mouse cells can translate in human studies [101].

The same group also demonstrated using a non-integrative episomal reprogramming approach, the in vitro generation of endodermal progenitor cells from neonatal and adult human skin fibroblasts. The group was then able to identify conditions that enabled this direct differentiation, initially into progenitors of posterior foregut, and then into pancreatic endodermal progenitors [102].

### 5.4. Keratinocytes

Expanding the arsenal of adult cells that can be exploited as a cell source for producing functional endocrine pancreatic cells, Mauda-Havakuk et al. were able to demonstrate that human ectoderm derived keratinocytes can transdifferentiate into β-cells that can secrete insulin in response to elevated glucose concentrations by ectopic expression of PDX1, and to lesser extent other transcription factors. Using lineage tracing for KRT-5 promoter activity, they presented evidence that insulin-positive cells are generated within a very short time frame (5 to 7 days) from ectoderm derived keratinocytes with insulin production in 12 ± 8% of reprogrammed cells [103]. Table 1 describes number of reprogramming models utilized for generation of insulin producing β-like cells.

## 6. Conclusions and Future Perspectives

Despite the vast potential of recent advances in cellular reprogramming and trans-differentiation of insulin producing cells, many issues still need refining in the coming decades. These include the efficiency of the cell trans-differentiation protocols, their cost-effectiveness for large-scale differentiation and whether reprogrammed cells can sustain their new differentiation state, or whether they potentially return to their early fate. Furthermore, whether transdifferentiated β-cells can respond adequately to the multitude of physiological stimuli required to maintain metabolic equilibrium and upon increased metabolic demand. Lastly, are the reprogrammed cells able to evade the recurrent auto-immune attacks, particularly in the context of allogenic cell transplants, and might cell transplants possess increased oncogenic potential resulting from incomplete epigenetic conversion? Several technologies have been proposed to attend to the immune responses to and blood supply for transplants, including the use of physical shielding by encapsulating the reprogrammed cells in a device that allows for insulin and nutrients to efficiently cross through the membrane while blocking cells from trespassing. Other biological interventions include either modifying host immune system, using antibodies to block the immune reactions or gene editing of cells used for transplant.

Furthermore, the safe implementation of viral reprogramming technologies to the human setting need to be further assessed. The small molecules approach for reprogramming are more intriguing than virus-based methods, but they are very complex to develop for the regulation of a number of transcription factors. Moreover, impeding to clinical translation of the reprogramming approach still, is the relatively limited conversion rate into functional bone fide β-cells and presence of unwanted cells in the final product

A paradigm shift has been made toward understanding fundamental β-cell biology, development and reprogramming over the last 20 years. Although a plethora of cell sources have been suggested as a starting material en route to successful reprogramming, some cells may fail. Thus, it is difficult to speculate which cells will reach the finish line and lead to a replacement therapy for diabetes.

In conclusion, we have witnessed a remarkable two decades of significant progress in the methods of creating cellular transplant for the cure of diabetes. Current clinical trials for T1DM are still using hESC-derived pancreatic progenitors as a surrogate for cadaveric material, but the debate over whether to transplant a more mature cell population more similar to human islets is still ongoing. Hepatic, gastrointestinal and pancreatic exocrine cells, which are derived from common endodermal progenitor cells, have the potential to take the lead as cell sources for the development of a therapeutic product. Identical developmental transcription mechanisms and regulatory networks, analogous chromatin landscapes, physical proximity to the injured pancreas and a minimal need for epigenomic rearrangement are all tempting to conclude that these functionally related cells may yield more robust reprogramming outcomes, and offers a unique promise for cell replacement therapies for diabetes.

## Figures and Tables

**Table 1:** Reprogramming approach of various cell types into insulin producing β-like cells (selected experimental models). Abbreviations: Adeno-associated virus (AAV), Alloxan (ALX); Lentiviral vector (LV); MAF BZIP Transcription Factor A (MAFA); MAF BZIP Transcription Factor B (MAFB); Myelin Transcription Factor 1 (MYT1); Neurogenin-3 (NGN3); Neurogenic differentiation 1 (NEUROD1); Non-obese diabetic (NOD) mice; the paired/homeodomain transcription factor (PAX4); Paired box domain 6 (PAX6); Insulin promoter factor 1 (PDX1); PDX1, MAFA, and NGN3 (PMN); TGFβ-induced factor homeobox 2 (TGIF2); 5-AZA 5-azacytidine.

Cell source	Origin	Reprogramming Tool	Model	Results	Reference
**Pancreatic Cell Sources**					
Alpha cells	Endoderm	Adeno-associated virus (AAV) carrying Pdx1 and MafA expression cassettes	ALX-induced diabetes and in autoimmune non obese diabetic (NOD) mice (in vivo)	-Alpha-cell-derived insulin+ cells have a similar expression profile to normal beta cells. -Prolonged normalization of blood glucose in hyperglycemic NOD mice for 4 months.	[34]
Acinar cells	Endoderm	Transcription factors overexpression Pdx1, MafA and Ngn3 (PMN cocktail)	Adult (Rag−/−), NOD mice (>2 month). (in vivo)	-Reprogrammed β-cells do not organize into islet structures. -New β-cells do not express exocrine genes. -Relatively fast speed direct conversion with the first insulin+ cells appear at day 3, and with efficiency of up to 20%.	[43]
Ductal epithelial cells	Endoderm	Ngn3 overexpression combined with modulation of the Delta-Notch signaling and addition of pancreatic endocrine transcription factors (Myt1, MafA and Pdx1)	Adult Human Duct Cells (in vitro)	-10% of full duct-to-endocrine reprogramming achieved.	[63]
**Extra-pancreatic cell sources**					
Hepatic cells	Endoderm	Expression of TGFβ-induced factor homeobox 2 (TGIF2), both ex-vivo and in-vivo	Murine adult primary or BAML hepatocytes (ex-vivo), mice models (in-vivo)	-Primary hepatocytes transduced with LV-TGIF2 formed pancreatic organoid structures. -AAV.TGIF2-injected mice displayed reduced blood glucose levels for 2 months.	[80]
Biliary tree, gallbladder, and cystic ducts	Endoderm	Adenoviral-mediated expression of transcription factors Pdx1, MafA, Neurog3, and Pax6	Primary cultures of human gallbladder and cystic duct cells (in vitro)	-Scalable in vitro expansion. -Insulin protein production (as measured by C-peptide) was found on day 5 and lasted for 3 months.	[81]
Intestinal cells	Endoderm	Transient intestinal expression of Pdx1, MafA, and Ngn3 (PMN) in the intestinal crypts	Mice (in vivo), human intestinal organoids	-Intestinal neo-islets were generated which are glucose-responsive and able to ameliorate hyperglycaemia in mice model of diabetes.	[92]
Stomach antral cells	Endoderm	Reprogramming of antral gastric cells with the PMN cocktail (PNM)	Mice (in vivo), Antral stomach and duodenal organoids were derived from young adult mice (1–2 months)	-Suppressed hyperglycaemia in a diabetic mouse model for at least 6 months and can regenerate rapidly after ablation.	[94]
Fibroblasts	Mesoderm	18 h of exposure of DNA methyltransferase inhibitor 5 azacytidine (5-AZA) followed by a three-step protocol for the induction of endocrine pancreatic differentiation that lasted 36 d	Adult human dermal fibroblasts (in vitro)	-Conversion of 35 ± 8.9% of fibroblasts into insulin producing cells at end of treatment protocol. -Converted cells were able to protect recipient mice against streptozotocin-induced diabetes, restoring a physiological response to glucose tolerance tests.	[98]
Keratinocytes	Ectoderm	Ectopic expression of Pdx1, NeuroD1, Ngn3 with high glucose concentration in culture	Cell culture of human keratinocytes (in vitro)	-Insulin production occurred in short time (5 to 7 days) with insulin pro-duction in 12 ± 8% of reprogrammed cells -More efficient than induced pluripotent cells (iPSC) which typically takes >20 days with an efficiency of 0.05–0.1%; or 3.3% in clonal cells	[103]
